# Isolated Chronic Tophaceous Gout of the Elbow: A Rare Presentation of Olecranon Bursopathy Requiring Surgical Excision

**DOI:** 10.7759/cureus.90345

**Published:** 2025-08-17

**Authors:** Sujith Shahul, Samreen Rafiuddin, Gautham Anil Nair, Tamer Farouk Hussein Elsaid, Habib Alismaily

**Affiliations:** 1 Orthopedics and Traumatology, Rashid Hospital, Dubai, ARE; 2 Orthopedics and Traumatology, RAK Medical Health &amp; Sciences University, Ras Al Khaimah, ARE

**Keywords:** chronic bursitis, excision, excision and biopsy, hyperuricaemia, tophaceous gout

## Abstract

Tophaceous gout is a chronic sequela of hyperuricemia, typically and predominantly affecting peripheral joints. However, isolated elbow involvement of the olecranon with a large tophaceous ulcer is uncommon. We report a rare case of chronic tophaceous olecranon bursopathy in a 48-year-old male patient with poorly controlled hyperuricemia, highlighting the diagnostic, imaging, and surgical aspects of this condition. The histopathological examination confirmed the presence of urate crystal tophi devoid of any malignant features.

## Introduction

Gouty arthritis is a metabolic disorder that occurs due to hyperuricemia which is characterized by the deposition of monosodium urate (MSU) crystals in joints, most commonly in the first metatarsophalangeal joint. Gout has a marked increase in prevalence in patients with chronic kidney disease, hypertension, metabolic syndrome, and diabetes. About 1% of the Western countries’ population is affected by gout [1]. Tophi are presentations of nodular masses of MSU in patients with untreated and chronically uncontrolled gout [2]. Typically, if untreated, gout transforms through four clinical stages: asymptomatic hyperuricemia, acute gout, intercritical or interval gout, and chronic tophaceous gout. Despite gouty tophi being seen as a chronic ailment, tophi itself may possibly signal the beginning of the disease [3].

## Case presentation

**Figure 1:**
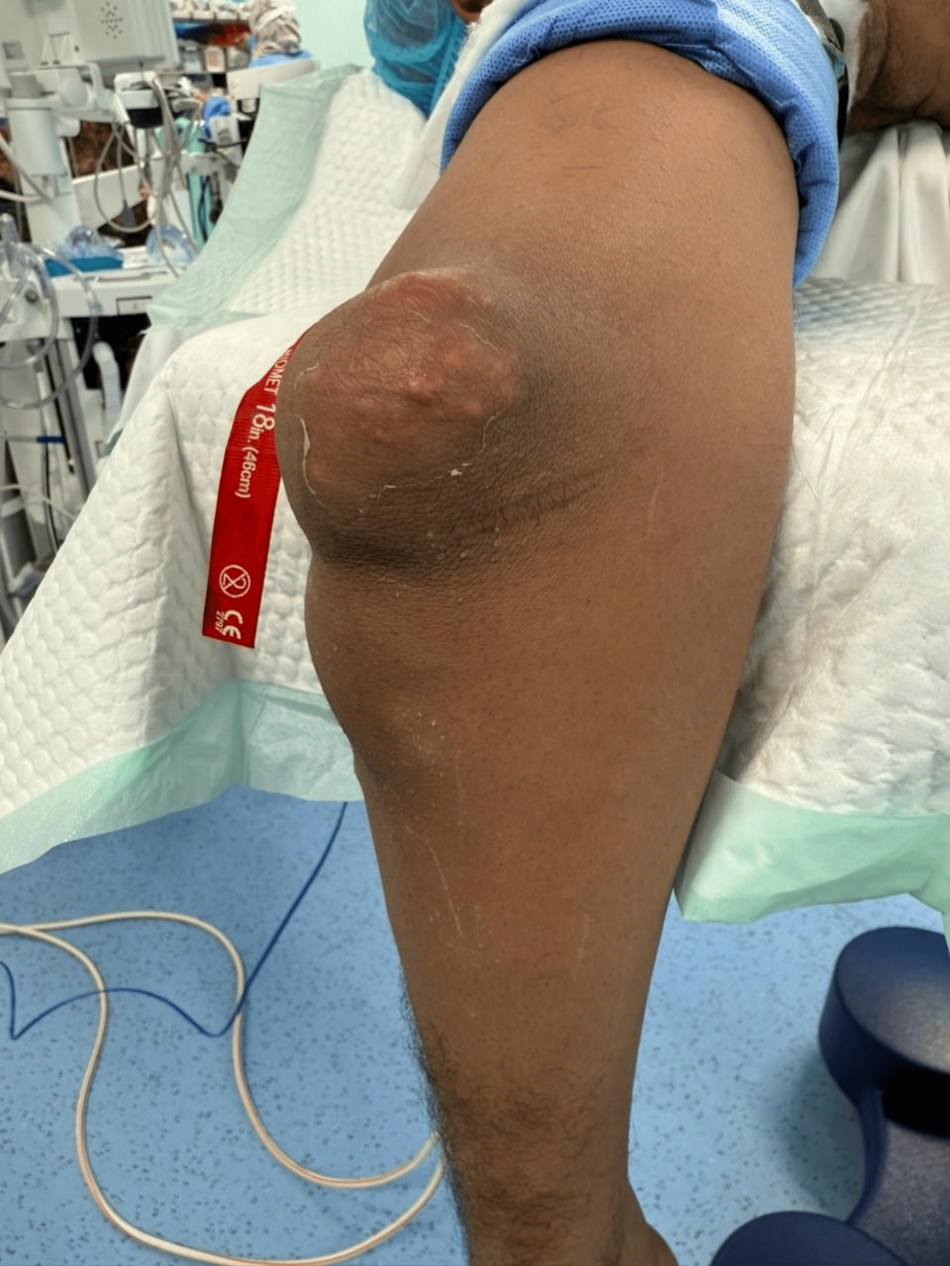
The patient upon presentation with edema and nodules at the left olecranon (side view)

**Figure 2:**
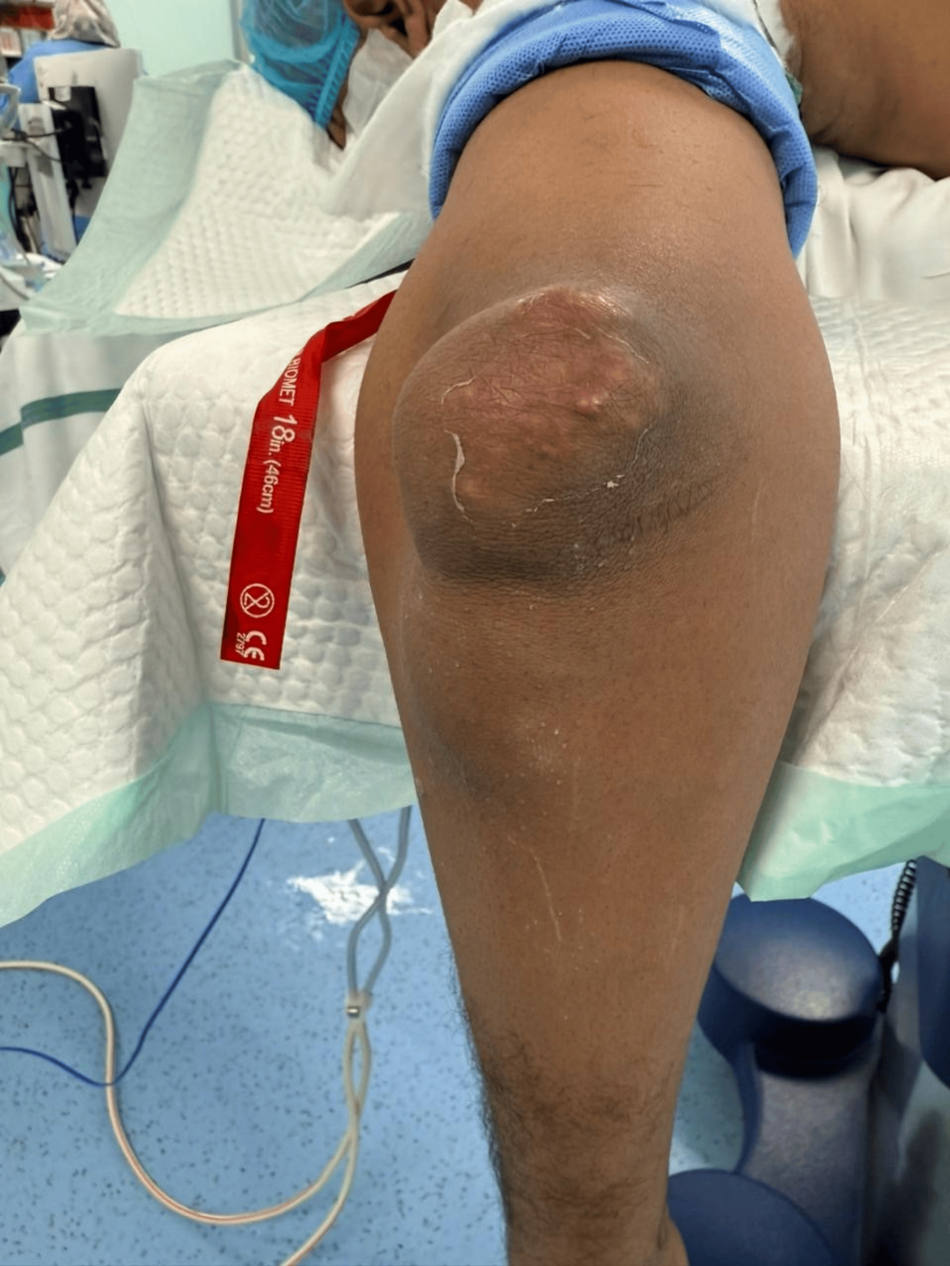
The patient upon presentation with edema and nodules at the left olecranon (front view). Lesion is firm with slight erythema and central chalky-white material indicating urate crystal deposition

A 48-year-old male patient, with a known case of chronic gout and hypertension, presented with a large tender swelling over his left elbow. The patient is a chronic smoker and alcoholic with a longstanding history of polyarticular gout attacks involving multiple joints such as the shoulder, knee, ankle, and toe. He was on febuxostat 80 mg and allopurinol 100 mg since December 2022 due to his history of gouty attacks. He was also put on colchicine 0.6 mg for his acute gout attacks. Clinical examination revealed a firm and nonfluctuant swelling (Figures 1, 2) over the olecranon region without any accompanying systemic symptoms such as fever or generalized inflammatory markers.

**Figure 3:**
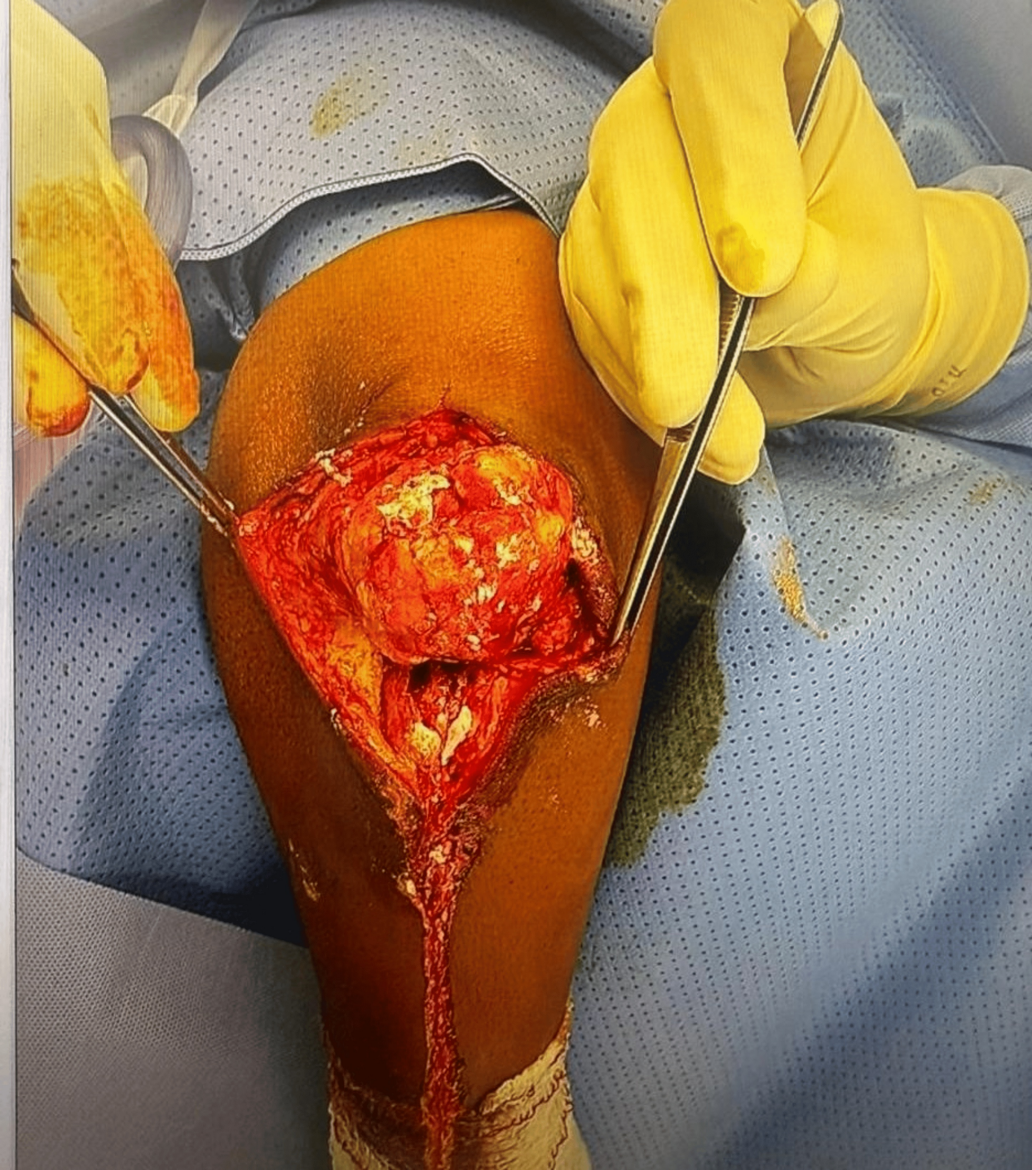
Intraoperative view showing excision of a large tophaceous mass from the olecranon region (front view). The lesion is surrounded by inflammatory and fibrotic tissue with visible urate crystal deposits infiltrating the subcutaneous and bursal planes

Initial musculoskeletal ultrasound showed hyperechoic deposits with no active Doppler signals within the olecranon bursa. No synovial hypertrophy or effusion was noted. The patient's C-reactive protein (CRP) and white blood cell (WBC) levels were significantly elevated (CRP: 213.3 mg/L; WBC: 13.5-18.4 x 109/L), indicating inflammation. Despite these markers, aspiration was not performed due to a lack of fluid presence. The surgical excision of the tophaceous mass (Figure 3) was successfully performed on December 1, 2024, under sterile operative conditions. The decision for operation was based on the ulcer's size and chronicity.

**Figure 4:**
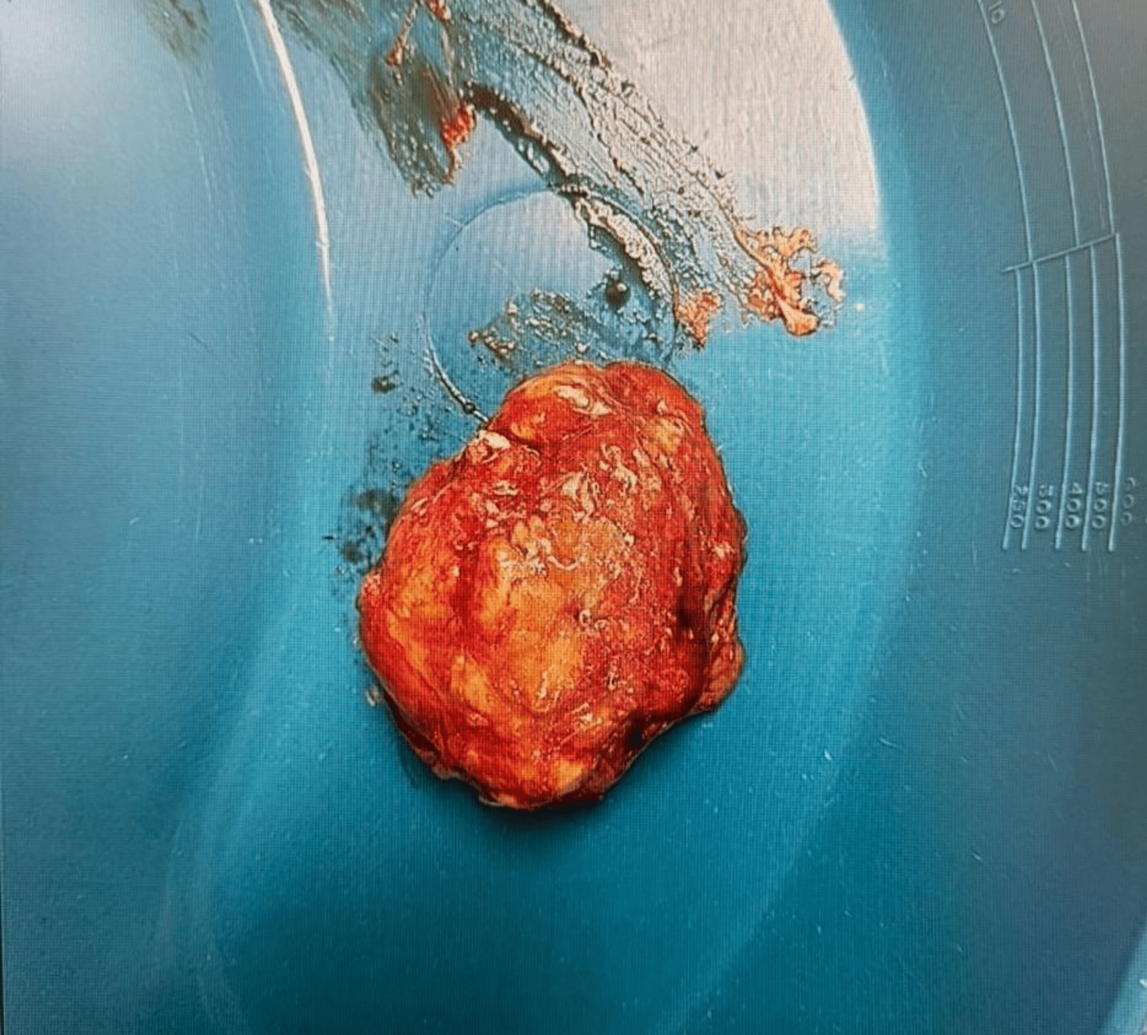
Gross specimen post-excision (5.0 x 4.0 x 4.0 cm) revealing a well encapsulated, lobulated, chalky, and urate deposit consistent with tophaceous gout mass

Histopathological analysis of the excised tissue (Figure 4) revealed acellular amorphous material with multinucleated giant cells, a hallmark of gouty tophus. The excised mass measured approximately 5.0 x 4.0 x 4.0 cm, with a chalky-white lobular appearance.

**Figure 5:**
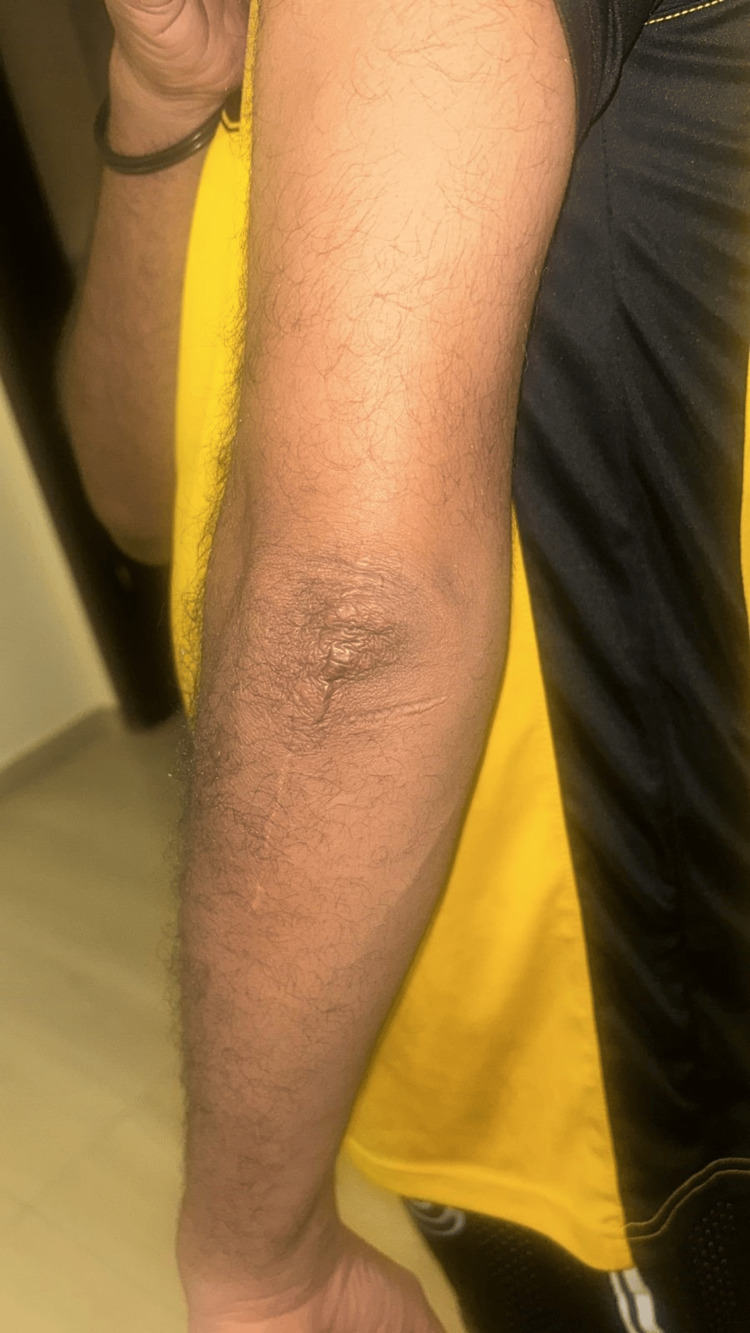
Six-month postoperative anterior view demonstrating a well-healed surgical scar and no residual swelling or inflammation

**Figure 6:**
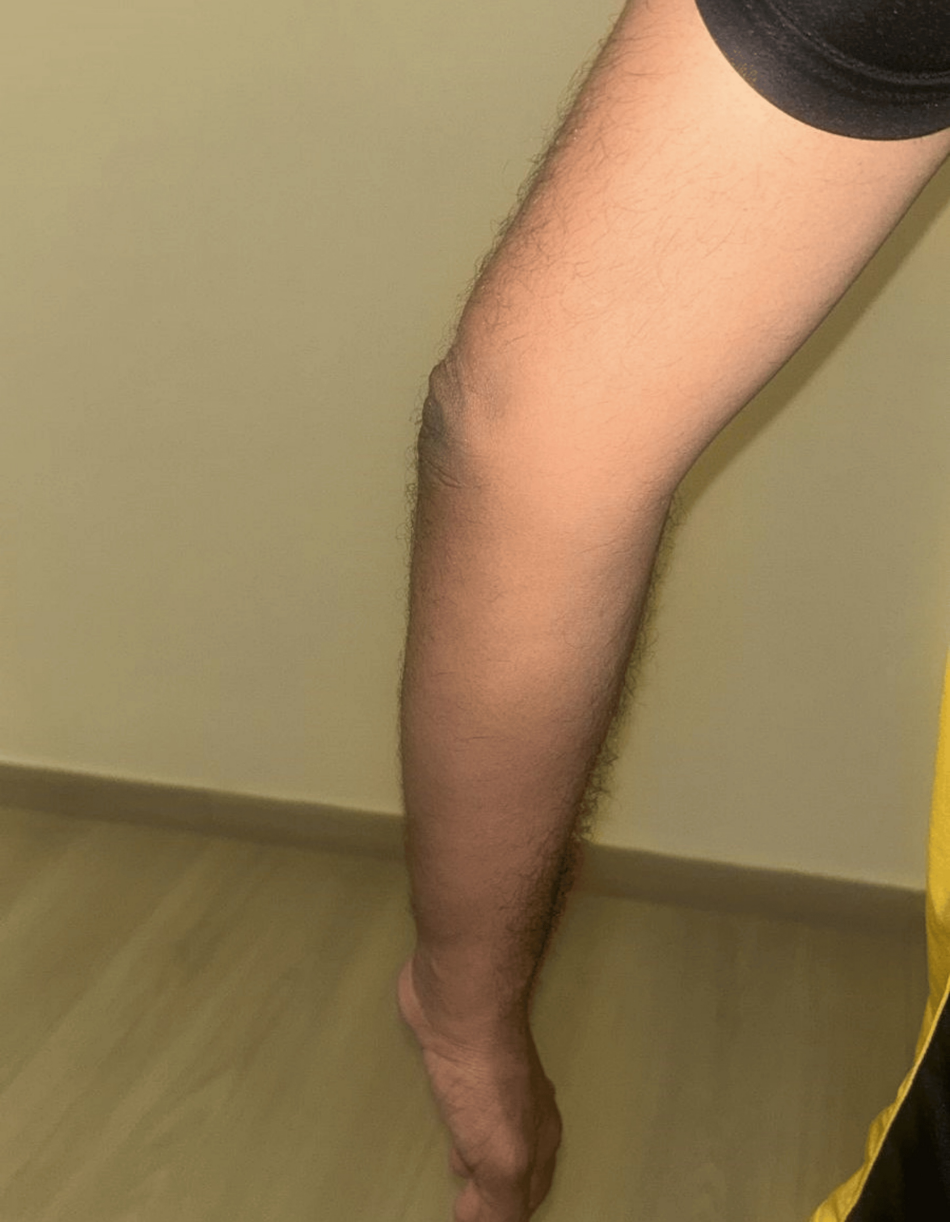
Lateral view six months postop demonstrates complete restoration of elbow contour indicating favorable surgical outcome

Postoperative recovery was uneventful with no complications, and urate-lowering therapy was reinforced (Figures 5, 6). The patient was advised on long-term dietary control and follow-up.

Prior to surgery, the patient's serum uric acid concentration was 8.6 mg/dL, which is well above the target treatment goals in patients with tophaceous gout. In the months prior to surgery, the urate concentrations were chronically high (>9-10 mg/dL), and biochemical control was never achieved below the target of <6 mg/dL and <5 mg/dL in patients with tophi as per the international guidelines.

The sustained hyperuricemia is directly related to the ongoing case of chronic tophaceous gout, contributing to the ongoing deposition of MSU crystals and thus lobulated, firm tophi around and inside the articulating structures. The high uric acid concentration prior to surgery reflects the patient's current active crystal burden at presentation and warrants discussion regarding surgical excision of the olecranon tophus, given his degree of recurrent inflammation, functional impairment, and visible deformity. Long-term management should focus on proactive and intensive lower urate targets, routine biochemical assessment, and detailed patient education to prevent recurrence and the risk of further joint damage.

## Discussion

Tophaceous involvement of the elbow joint, in the absence of other severe deformities in the elbow or elsewhere in the joints, is infrequent. In this patient, after years of poorly controlled gout, there were hypertrophic changes and bursal swelling because of the elevated serum uric acid level that can be seen in gouty arthritis. Acute tophaceous gout, a component of inflammatory arthritis, is due to a relatively high burden of crystals that may require surgical intervention for visible deformity based on extremity of arthropathy [4].

Considering the significant increase in the prevalence of gout, the importance of analyzing its influence on health-related quality of life, productivity, and healthcare resource utilization is on the rise. In a cross-sectional survey performed with the US and EU National Health and Wellness Surveys in 2012, they discovered that out of n = 620 (338 in the US and 282 across France, Germany, and the UK), 12.3% (n = 76) reported the presence of tophi with acute flares [5]. In a microscopic lens, tophi presents itself as a chronic, foreign body-type granuloma composed of dense MSU crystal clusters encased in layers of inflammatory immune cells and connective tissue. Evidence suggests that neutrophil interactions with MSU crystals, especially during the formation of neutrophil extracellular traps, offer a key control point in both building and maintaining tophi. Chronic MSU crystal deposition in the olecranon bursa can also ignite robust inflammation that can lead to bursal swelling and potential ulceration. The development of health concerns, including structural joint damage and increased mortality risk, is increased due to tophi in people with gout [6].

The impacts of gouty arthritis with tophi affect numerous daily lifestyle aspects, ranging from pain, restricted joint motion, joint deformities, and severe complications such as ulcerations and infections. According to a case-controlled study conducted in Wenzhou Medical University, elderly patients who demonstrated a prolonged ulcer duration and larger ulcer size were found to be more vulnerable to infection [7]. In this case, poor control of gout for many years led to significant hypertrophic and inflammatory changes to the elbow region. Diagnosis may be delayed in the absence of classical gouty flares. Imaging, including ultrasound, is an important diagnostic method for identifying crystal deposits in tendons and bursae. Definitive diagnosis is mainly decided by histopathological examination showing amorphous eosinophilic material and giant cells, which is considered a hallmark of diagnosis, and was also demonstrated in this case [8].

To maximize the effective management of gout flares, nonsteroidal antiinflammatory drugs, colchicine, or glucocorticoids (oral, intraarticular, or intramuscular) are greatly recommended [9]. Timely surgical intervention is key to preventing potential risks of infection and nerve compression. Ultimately, surgical debridement of tophaceous material is the preferred treatment regimen when pharmacologic therapies have not resolved local symptoms or when there is an atypical presentation of the mass [10].

## Conclusions

This case highlights the rare occurrence of isolated chronic tophaceous gout of the elbow. Such atypical presentations in patients with long-standing and inadequately managed hyperuricemia necessitate the need for special care from the clinician to avoid misdiagnosis and prevent adverse complications. Surgical removal of tophi remains the treatment of choice when medical therapy fails or complications arise. Early recognition and a multidisciplinary approach are apt to prevent irreversible joint damage in advanced gouty arthropathy to achieve the best clinical outcomes.

## References

[1] Terkeltaub R (2009). Gout. Novel therapies for treatment of gout and hyperuricemia. Arthritis Res Ther.

[2] Sriranganathan MK, Vinik O, Falzon L, Bombardier C, van der Heijde DM, Edwards CJ (2014). Interventions for tophi in gout: a Cochrane systematic literature review. J Rheumatol Suppl.

[3] Koley S, Salodkar A, Choudhary S, Bhake A, Singhania K, Choudhury M (2010). Tophi as first manifestation of gout. Indian J Dermatol Venereol Leprol.

[4] Dalbeth N, Merriman TR, Stamp LK (2016). Gout. Lancet.

[5] Khanna PP, Nuki G, Bardin T (2012). Tophi and frequent gout flares are associated with impairments to quality of life, productivity, and increased healthcare resource use: results from a cross-sectional survey. Health Qual Life Outcomes.

[6] Chhana A, Dalbeth N (2015). The gouty tophus: a review. Curr Rheumatol Rep.

[7] Xu J, Zhu Z, Zhang W (2018). Clinical characteristics of infectious ulceration over tophi in patients with gout. J Int Med Res.

[8] Zhang W, Doherty M, Bardin T (2006). EULAR evidence based recommendations for gout. Part II: management. Report of a task force of the EULAR Standing Committee for International Clinical Studies Including Therapeutics (ESCISIT). Ann Rheum Dis.

[9] FitzGerald JD, Dalbeth N, Mikuls T (2020). 2020 American College of Rheumatology guideline for the management of gout. Arthritis Care Res (Hoboken).

[10] Richette P, Doherty M, Pascual E (2017). 2016 updated EULAR evidence-based recommendations for the management of gout. Ann Rheum Dis.

